# Enhancing the Lasso Approach for Developing a Survival Prediction Model Based on Gene Expression Data

**DOI:** 10.1155/2015/259474

**Published:** 2015-06-03

**Authors:** Shuhei Kaneko, Akihiro Hirakawa, Chikuma Hamada

**Affiliations:** ^1^Department of Management Science, Graduate School of Engineering, Tokyo University of Science, 1-3 Kagurazaka, Shinjuku-ku, Tokyo 162-8601, Japan; ^2^Biostatistics and Bioinformatics Section, Center for Advanced Medicine and Clinical Research, Nagoya University Graduate School of Medicine, 65 Tsurumai-cho, Showa-ku, Nagoya 466-8560, Japan

## Abstract

In the past decade, researchers in oncology have sought to develop survival prediction models using gene expression data. The least absolute shrinkage and selection operator (lasso) has been widely used to select genes that truly correlated with a patient's survival. The lasso selects genes for prediction by shrinking a large number of coefficients of the candidate genes towards zero based on a tuning parameter that is often determined by a cross-validation (CV). However, this method can pass over (or fail to identify) true positive genes (i.e., it identifies false negatives) in certain instances, because the lasso tends to favor the development of a simple prediction model. Here, we attempt to monitor the identification of false negatives by developing a method for estimating the number of true positive (TP) genes for a series of values of a tuning parameter that assumes a mixture distribution for the lasso estimates. Using our developed method, we performed a simulation study to examine its precision in estimating the number of TP genes. Additionally, we applied our method to a real gene expression dataset and found that it was able to identify genes correlated with survival that a CV method was unable to detect.

## 1. Introduction

In the past decade, researchers have predicted survival in a cancer patient based on gene expression data [1–4]. Revealing the relationship between gene expression profiles and the time to an event of interest (e.g., overall survival, metastasis-free survival) can improve treatment strategies and establish accurate prognostic markers. The Cox proportional hazard model is the most popular method for relating covariates to survival times [5]. However, due to the high dimensionality of gene expression data (i.e., the number of genes expressed exceeds the number of patients), it is not possible to take an estimation approach based on the Cox log partial likelihood. To overcome this problem, a penalized estimation approach, which includes a shrinkage estimation of coefficients, is frequently taken [6–8].

In penalized estimation approaches, the least absolute shrinkage and selection operator (lasso) [9, 10] is often used because of its attractive ability to simultaneously select the genes correlated with survival and estimate the coefficients in the Cox model. The lasso shrinks most of the coefficients towards zero exactly by adding *L*_1_ norm to the Cox log partial likelihood, and the amount of shrinkage is dependent on the tuning parameter. The value of the tuning parameter is often determined by a cross-validation (CV), which maximizes the out-of-data prediction accuracy [11].

Several researchers have investigated the operating characteristics of the lasso. Goeman [12] used the lasso to analyze a publicly available gene expression dataset, obtained from the articles of van't Veer et al. [2] and van de Vijver et al. [3] in which a 70-gene signature for prediction of metastasis-free survival in breast cancer patients had been established. This data included 295 patients with 4919 genes that were prescreened from 24,885 genes based on the quality criteria in van't Veer et al.'s work [2]. The lasso selected 16 genes with which to develop a prediction model of overall survival when using the tuning parameter that was determined using a CV. Goeman [12] also conducted ridge regression using all 4919 genes to develop a model by adding *L*_2_ norm to the Cox log partial likelihood. The prediction accuracy of the lasso and ridge regression were compared, and the ridge regression with 4919 genes slightly outperformed the lasso with 16 genes. Goeman [12] concluded that the lasso potentially passes over genes that are correlated with survival in order to develop a simple prediction model. Bøvelstad et al. [7] reached the same conclusion in a review of the survival prediction methods available for analyzing breast cancer gene expression datasets.  Table 1 summarizes a typical result of gene selection by the lasso.

The CV method determines the value of the tuning parameter by considering the trade-off between the number of true positives (TP) and false positives (FP), and so the possibility of identifying false negatives (FN) cannot be eliminated. One solution for identifying more outcome-predictive genes is to monitor the number of TP in several values of the tuning parameter and, subsequently, determine its final value. In this study, we developed a method for estimating the number of TP for a series of values of the tuning parameter. We assumed a mixture distribution with components of TP and FP for the lasso estimates, and these could be used to estimate the number of TP and FP. It is possible to generate the solution path that includes the lasso estimates for a series of values of the tuning parameter using the methods developed by Goeman [12]. Here, we proposed an algorithm to sequentially fit the mixture distribution for this solution path, and we used a simulation study to test the precision of the algorithm when estimating the number of TP. We further demonstrated the proposed algorithm using a well-known diffuse large B-cell lymphoma (DLBCL) dataset comprising overall survival of 240 DLBCL patients and gene expression data of 7399 genes [1].

## 2. Materials and Methods

### 2.1. Lasso in the Cox Proportional Hazard Model

The Cox proportional hazard model is the most popular method for evaluating the relationship between gene expression and time to an event of interest [5]. The hazard function of an event at time *t* for a patient *i*  (*i* = 1,…, *n*) with the gene expression levels **x**_*i*_ = (*x*_*i*1_,…, *x*_*ip*_)^T^ is given by

(1) $$\begin{array}{c} h\left(t\;|\;x_{i}\right)=h_{0}\left(t\right)\exp\left(x_{i}^{T}\beta \right), \end{array}$$

where **β** = (*β*_1_,…, *β*_*p*_)^T^ is a parameter vector and *h*_0_(*t*) is the baseline hazard, which is the hazard for the respective individual when all variable values are equal to zero. In the general setting where *n* > *p*, the coefficients are estimated by maximizing Cox log partial likelihood as follows:

(2) $$\begin{array}{c} l\left(\beta \right)=\overset{n}{\underset{i=1}{\sum }}\delta_{i}\left[x_{i}^{T}\beta -log\left\{\underset{r\in R\left(t_{i}\right)}{\sum }exp\left(x_{r}^{T}\beta \right)\right\}\right], \end{array}$$

where *δ*_*i*_ is an indicator, which is 1, if the survival time is observed, or 0, if censored. *R*(*t*_*i*_) is the risk set of the individuals at *t*_*i*_.

In the lasso for the high-dimensional setting where *n* < *p*, the coefficients are estimated by maximizing the following penalized likelihood function [9, 10]:

(3) $$\begin{array}{c} l_{p}\left(\beta ,\lambda \right)=l\left(\beta \right)-\lambda \overset{p}{\underset{j=1}{\sum }}\left|\beta_{j}\right|, \end{array}$$

where *λ* is the tuning parameter, which determines the amount of shrinkage.

### 2.2. Solution Path of the Lasso Estimates

Goeman [12] introduced a method to calculate the solution path of the lasso estimates as a function of *λ*, $\hat{\beta }(\lambda )$, which is based on the algorithm developed by Park and Hastie [13]. The method maximizes *l*_*p*_(**β**, *λ*) at a fixed *λ* based on a combination of gradient ascent optimization with the Newton-Raphson algorithm. $\hat{\beta }(\lambda )$ are calculated for *λ*_0_ > ⋯>*λ*_*k*_ > ⋯>*λ*_*z*_ > 0 successively, starting from *λ*_0_ = max_*j*_∂*l*/∂*β*_*j*_|_*β*_*j*_=0_ (which gives $\hat{\beta }(\lambda_{0})=0$ because the value has zero gradients). *λ*_*z*_ is chosen arbitrarily but is often set to 0.05 × *λ*_0_ in analyses of gene expression data [14]. The lasso estimates at a current step are set to initial values for calculation of the subsequent step. Step length Δ_*k*_ = *λ*_*k*_ − *λ*_*k*+1_ is the minimum decrement to change the number of selected genes $m^{\left(k\right)}\;(=\#\{j;{\hat{\beta }}_{j}(\lambda_{k})\neq 0\})$; that is, only one gene is newly selected or excluded from *λ*_*k*_ to *λ*_*k*+1_.

### 2.3. Mixture Distribution for Estimating the Number of TP in the Lasso Estimates

To estimate the number of TP in the lasso estimates at a fixed value of *λ*, we assumed a mixture distribution developed in our previous study [15]. We introduced the mixture distribution based on the two features of the lasso: (i) the lasso selects at most *n* genes because of the nature of the convex optimization problem when *n* < *p* [16, 17] and (ii) in the Bayesian paradigm the lasso estimates are the posterior mode with the independent Laplace prior distribution *f*_*L*_(*β*_*j*_; 0,1/*τ*) = (*τ*/2)exp⁡(−*τ* | *β*_*j*_|), where *f*_*L*_(*y*; *a*, *b*) = 1/2*b*exp(−|*y* − *a* | /*b*) is the probability density function of Laplace distribution with location parameter *a* and scale parameter *b* [9]. Therefore, the mixture distribution assumed for the lasso estimates at *λ* was as follows:

(4) fβ^jλ;π0,πc,τ,μc,σc =npπ0fLβ^jλ;0,1τ+∑c=1CπcfNβ^jλ;μc,σc2  +1−npfLβ^jλ;0,ϵ,

where *π*_0_ and *π*_*c*_ are mixed proportions (*π*_0_ + ∑_*c*=1_^*C*^ *π*_*c*_ = 1); $f_{N}\left({\hat{\beta }}_{j}(\lambda );\mu_{c},\sigma_{c}^{2}\right)$ is the probability density function of the normal distribution with mean *μ*_*c*_  (≠0) and variance *σ*_*c*_^2^ in component *c*; *C* is the number of components, which is determined by model selection criteria; and *ϵ* is the constant value, which is boundlessly close to 0; for example, *ϵ* = 10^−8^. The unknown parameters, *π*_0_, *π*_*c*_, *τ*, *μ*_*c*_, and *σ*_*c*_, are estimated by maximizing the log-likelihood function of (4) by using the Newton-Raphson method.

The mixture distribution defined in (4) is formulated on the basis of the following concepts: since the lasso selects a maximum of *n* genes when *p* > *n*, the coefficients for *p* − *n* genes are exactly zero; therefore, (4) consists of 2 terms (*n*/*p* term and 1 − *n*/*p* term). In the *n*/*p* term, the Laplace distribution with location parameter 0 and scale parameter 1/*τ* was assumed to be the distribution for the FP on the basis of the lasso feature (ii) discussed above, while the *C* component normal distribution with location parameter *μ*_*c*_ and scale parameter *σ*_*c*_^2^ was assumed as the distribution for the TP. In the 1 − *n*/*p* term, the Laplace distribution with location parameter 0 and scale parameter *ϵ* was assumed as the distribution of *p* − *n* genes based on the aforementioned lasso feature (i).

The *f*_*L*_ with location parameter 0 and scale parameter 1/*τ* was assumed to be the distribution for the FP on the basis of lasso feature (i), discussed above. The *f*_*N*_ with location parameter *μ*_*c*_ and scale parameter *σ*_*c*_^2^ was assumed as the distribution for the TP. The *f*_*L*_ of the (1 − *n*/*p*) term was assumed as the distribution of *p* − *n* genes based on the aforementioned lasso feature (ii). Given a cut-off value *ζ* (>0), the estimated proportions of the FP and TP are the area under the estimated Laplace and normal distribution in the *n*/*p* term of (4), respectively, and can be written as follows:

(5) P^FP=π^0∫−∞−ζfLu;0,τ^−1du+∫ζ+∞fLu;0,τ^−1du,P^TP=∑c=1Cπ^c∫−∞−ζfNu;μ^c,σ^c2du+∫ζ+∞fNu;μ^c,σ^c2du.

**Figure 1:**
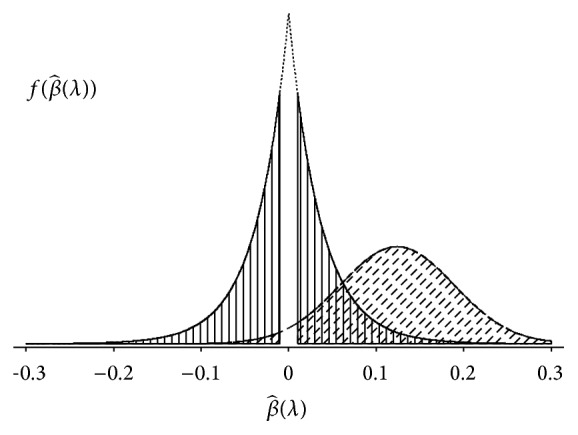
Illustration for estimating the number of FP and TP. The areas denoted by the vertical and diagonal lines are the proportion of FP and TP, respectively.

Figure 1 illustrates the calculation in (5) when the number of components, *C*, is 1. Using (5), the number of TP and FP was estimated by

(6) $$\begin{array}{c} \hat{FP}=\frac{{\hat{P}}_{FP}}{{\hat{P}}_{TP}+{\hat{P}}_{FP}}\times m, \end{array}$$

(7) $$\begin{array}{c} \hat{TP}=\frac{{\hat{P}}_{TP}}{{\hat{P}}_{TP}+{\hat{P}}_{FP}}\times m. \end{array}$$

### 2.4. Algorithm for Estimating Number of TP in a Series of Values  ***λ***

Here, we propose an algorithm to sequentially fit the mixture distribution in (4) to the solution path of the lasso estimates, which was described in Section 2.2. In this algorithm, we assumed that the number of TP changed when the newly selected or excluded gene from *λ*_*k*_ to *λ*_*k*+1_ was truly correlated to survival, based on the maximum log-likelihood of (4). First, we approximated ${\hat{P}}_{FP}\approx {\hat{\pi }}_{0}$ and ${\hat{P}}_{TP}\approx \sum_{c=1}^{C}{\hat{\pi }}_{c}$ in (5) by assuming a suitably small cut-off value *ζ* (≈0). We then obtained ${\hat{\pi }}_{0}=\hat{FP}/m$ and ${\hat{\pi }}_{c}={\hat{TP}}_{c}/m\;(c=1,\ldots ,C)$ from (6) and (7), respectively, where ${\hat{TP}}_{c}$ is an estimate of the number of TP in component *c*. For *k* = 1,…, *z*, the proposed algorithm was as follows.

*Step 1*

*Step 1.1*. In this step, we assumed that the newly selected or excluded gene from *λ*_*k*_ to *λ*_*k*+1_ was FP. *π*_0_ denotes the proportion of FP and is set as

(8) $$\begin{array}{c} \pi_{0}^{\left(k+1\right)}=\left\{\begin{array}{cc} \frac{{\hat{FP}}^{\left(k\right)}+1}{m^{\left(k+1\right)}}, & if\;m^{\left(k+1\right)}=m^{\left(k\right)}+1,\\ \frac{{\hat{FP}}^{\left(k\right)}-1}{m^{\left(k+1\right)}}, & if\;m^{\left(k+1\right)}=m^{\left(k\right)}-1. \end{array}\right. \end{array}$$

For the other components, *c*  (*c* = 1,…, *C*), set $\pi_{c}^{\left(k+1\right)}={\hat{TP}}_{c}^{\left(k\right)}/m^{\left(k+1\right)}$.

*Step 1.2*. Given $\hat{\beta }(\lambda_{k+1})$ and *π*_0_^(*k*+1)^,…, *π*_*C*_^(*k*+1)^, calculate the maximum log-likelihood of (4), *L*_0_^(*k*+1)^.

*Step 2*

*Step 2.1*. Set *c* = 1.

*Step 2.2*. In this step, we assumed that the newly selected or excluded gene from *λ*_*k*_ to *λ*_*k*+1_ was TP. For the component *c*, set

(9) $$\begin{array}{c} \pi_{c}^{\left(k+1\right)}=\left\{\begin{array}{cc} \frac{{\hat{TP}}_{c}^{\left(k\right)}+1}{m^{\left(k+1\right)}}, & if\;m^{\left(k+1\right)}=m^{\left(k\right)}+1,\;\\ \frac{{\hat{TP}}_{c}^{\left(k\right)}-1}{m^{\left(k+1\right)}}, & if\;m^{\left(k+1\right)}=m^{\left(k\right)}-1. \end{array}\right. \end{array}$$

For the other components, set $\pi_{0}^{\left(k+1\right)}={\hat{FP}}^{\left(k\right)}/m^{\left(k+1\right)}$ and $\pi_{d}^{\left(k+1\right)}={\hat{TP}}_{d}^{\left(k\right)}/m^{\left(k+1\right)}\;(d=1,\ldots ,C;d\neq c)$.

*Step 2.3*. Given $\hat{\beta }(\lambda_{k+1})$ and *π*_0_^(*k*+1)^,…, *π*_*C*_^(*k*+1)^, calculate the maximum log-likelihood of (4), *L*_*c*_^(*k*+1)^.

*Step 2.4*. Set *c* = *c* + 1. Repeat Steps 2.2 and 2.3 until *c* = *C*.

*Step 3*. In this step, we determined whether the newly selected or excluded gene from *λ*_*k*_ to *λ*_*k*+1_ was TP or FP based on the maximum log-likelihood which was calculated in Steps 1.2 and 2.3. If *L*_0_^(*k*+1)^ was the largest in *L*_*c*_^(*k*+1)^  (*c* = 0,…, *C*), we assumed that the newly selected or excluded gene was FP; if not, we assumed that it was TP. Therefore, calculate *C*_max_ = argmax_*c*∈{0,1,…,*C*}_⁡*L*_*c*_^(*k*+1)^. If *C*_max_ = 0, update ${\hat{FP}}^{\left(k\right)}$ as follows:

(10) $$\begin{array}{c} {\hat{FP}}^{\left(k+1\right)}=\left\{\begin{array}{cc} {\hat{FP}}^{\left(k\right)}+1, & if\;m^{\left(k+1\right)}=m^{\left(k\right)}+1,\\ {\hat{FP}}^{\left(k\right)}-1, & if\;m^{\left(k+1\right)}=m^{\left(k\right)}-1. \end{array}\right. \end{array}$$

If *C*_max_ > 0, update ${\hat{TP}}_{C_{max}}^{\left(k\right)}$ as follows:

(11) $$\begin{array}{c} {\hat{TP}}_{C_{max}}^{\left(k+1\right)}=\left\{\begin{array}{cc} {\hat{TP}}_{C_{max}}^{\left(k\right)}+1, & if\;m^{\left(k+1\right)}=m^{\left(k\right)}+1,\\ {\hat{TP}}_{C_{max}}^{\left(k\right)}-1, & if\;m^{\left(k+1\right)}=m^{\left(k\right)}-1. \end{array}\right. \end{array}$$

Here, calculate the estimated TP at *k* + 1 by ${\hat{TP}}^{\left(k+1\right)}=\sum_{c=1}^{C}{\hat{TP}}_{c}^{\left(k+1\right)}$.

## 3. Results

### 3.1. Simulation Study

We performed a simulation study to examine the precision of our estimated TP. In this study, the number of patients, *n*, was set to 200. The number of genes, *p*, was set to 1000, which included the *p*_1_  (=5 or 30) outcome-predictive genes that are randomly chosen from *p* genes in each simulation. The coefficient for gene *j*  (*j* = 1,…, *p*), *β*_*j*_, was set to 1.5 for the *p*_1_ outcome-predictive genes and 0 for the remaining *p* − *p*_1_ none-outcome-predictive genes. We set *λ*_*z*_ to 5 and the number of components, *C*, to 1 throughout (although *C* was determined using a model selection criterion in practice). The gene expression levels for patient *i*, **x**_*i*_, were generated from the multivariate normal distribution with mean vector 0 and covariance matrix Σ so that the variance was 1 and the correlation *ρ*(*x*_*ik*_, *x*_*il*_) = 0 or 0.5^|*k*−*l*|^ [18]. The survival time for patient *i* was generated based on the exponential model *t*_*i*_ = −log(*U*)/exp(**x**_*i*_^T^ **β**) where *U* is the uniform random variable between 0 and 1 [19]. In order to evaluate the precision of the estimated TP for various values of *λ*, we report a number of selected genes, including true TP, and estimated TP and FP, for *λ*_*k*_  (*k* = 5,10,50,100,150).

Table 2 shows the average of *λ*, a number of selected genes, true TP, and estimated TP and FP, through 1000 repeats. We observed that the precision of estimated TP varied depending on the value of both *p*_1_ and *k* (see Table 2). When *p*_1_ = 5, the precision of the estimates was sufficient for *k* = 10, 50, 100, and 150, while TP was slightly underestimated for *k* = 5. However, when *p*_1_ = 30, the precision of the estimates was sufficient for *k* = 5, 10, and 150, while TP was overestimated for *k* = 50 and 100. For example, when *p*_1_ = 30, *ρ* = 0.5, and *k* = 100, the average number of true and estimated TP was 29.9 and 35.3, respectively. The values of *ρ* did not greatly affect the accuracy of the estimated TP.

### 3.2. Real Data Analysis

To illustrate how our proposed algorithm could be used to determine *λ*, we applied it to the DLBCL dataset, comprising survival of 240 DLBCL patients and gene expression data from 7399 genes [1]. In the gene expression data from the 240 patients, we identified 434 genes with complete sets of gene expression values; all other genes had missing expression values, with an average of 24.7 missing values per gene. Here, we used 0.0 as the missing expression value for descriptive purposes. Similar to Rosenwald et al. [1], we divided the data into two: training data consisting of 160 patients and validation data consisting of 80 patients.

For the training data, we obtained the solution path of the lasso estimates; $\hat{\beta }(\lambda_{k})\;(k=0,1,\ldots ,z)$. *λ*_0_ = 72.5 was calculated as described in Section 2.2. We set *λ*_*z*_ = 3.625 ( = 0.05 × *λ*_0_) according to Simon et al. [14].

We applied our proposed algorithm to the obtained solution path. We assumed three mixture distributions on the lasso estimates with *C* = 1, 2, or 3 and compared their goodness of fit for the $\hat{\beta }(\lambda_{k})\;(k=0,1,\ldots ,z)$ by the Akaike information criterion (AIC). As a result, we chose *C* = 1 because it had the best AIC for all *λ*_*k*_  (*k* = 0,1,…, *z*).

**Figure 2:**
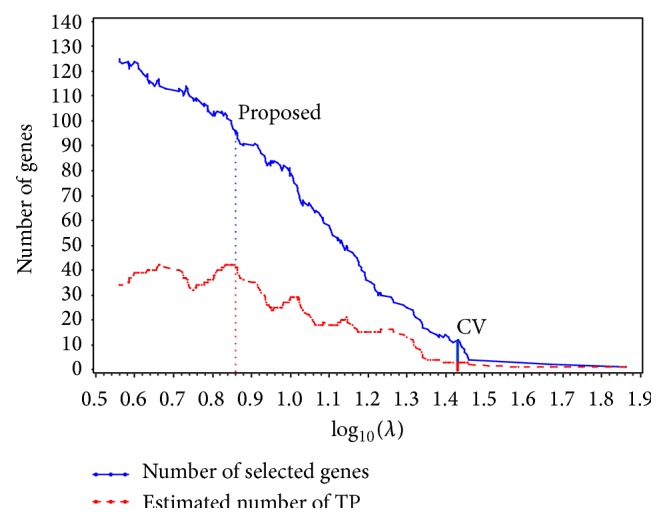
Trace plot of number of selected genes and estimated number of true positives (TP) produced by applying the proposed algorithm to the training data from the diffuse large B-cell lymphoma (DLBCL) dataset. We determined *λ* = 7.19 (log_10_ = 0.86) as the optimum *λ* based on the estimated number of TP. Using cross-validation (CV), we determined *λ* = 27 (log_10_ = 1.43) as the optimum *λ*.

Figure 2 shows the estimated number of TP in a series of values of *λ*. We found that the lasso selected at most 42 TP, with the number of selected genes at 96, when *λ* = 7.19 (= 0.86 as log_10_). Therefore, we selected *λ* = 7.19 as the optimum *λ*, and the estimated mixture distribution for the value of *λ* was as follows:

(12) fβ^j7.19=16073990.57×fLβ^j7.19;0,0.11+0.4300000000×fNβ^j7.19;0.03,0.112 +72397399fLβ^j7.19;0,10−8.

In order to identify the 42 TP from the 96 selected genes, we arranged the 96 in descending order of $\left|{\hat{\beta }}_{j}\right|$ and identified the first 42 listed genes with a cut-off value *ζ* = 0.084. Subsequently, the model that included these 42 genes is identified as the “42 TP-model.”

In comparison to the 42 TP-model, we performed CV. Briefly, the *K*-fold CV was given by

(13) $$\begin{array}{c} CV\left(\lambda \right)=\overset{K}{\underset{k=1}{\sum }}\left\{l\left({\hat{\beta }}_{\left(-k\right)}\left(\lambda \right)\right)-l_{\left(-k\right)}\left({\hat{\beta }}_{\left(-k\right)}\left(\lambda \right)\right)\right\}, \end{array}$$

where *l*_(−*k*)_(**β**) and ${\hat{\beta }}_{\left(-k\right)}$ are the log partial likelihood and the lasso estimate with left *k*th fold out, respectively. The optimal value of *λ* was obtained by maximizing CV(*λ*). On the basis of 5-fold CV, 12 genes were selected with *λ* = 27 (=1.43 as log_10_). Subsequently, the model including these 12 genes is identified as the “CV-model.” Notably, both the 42 TP-model with 42 genes and the CV-model with 12 genes selected 4 genes in common.  Table 3 shows the GenBank accession number and description for each of the 4 genes selected by both models.

We compared the prediction accuracy of the 42 TP-model and the CV-model using validation data consisting of 80 patients. For this data, we calculated 3 values that served as comparison criteria: *P* values for the log-rank test and prognostic index and the deviance. The 80 patients were categorized into 2 groups, the “better” and “worse” prognostic groups, using the boundary of the median of prognostic index ${\hat{\eta }}_{i}=x_{i}^{T}\beta$. The Kaplan-Meier curves between the 2 groups were compared with a log-rank test. Next, we calculated the *P* value for the parameter *α* multiplied by the prognostic index ${\hat{\eta }}_{i}$ in the Cox proportional hazard model $h(t_{i}|x)=h_{0}(t)exp\left(\alpha {\hat{\eta }}_{i}\right)$. Finally, the deviance was calculated by $-2\{l^{\left(validation\right)}\left({\hat{\beta }}_{training}\right)-l^{\left(validation\right)}(0)\}$, where $l^{\left(validation\right)}({\hat{\beta }}_{training})$ and *l* ^(validation)^(0) are the Cox log partial-likelihood function for the estimated coefficients by using the training data and zero vector 0, respectively. For each criterion, the lower value suggested better prediction accuracy.

**Figure 3:**
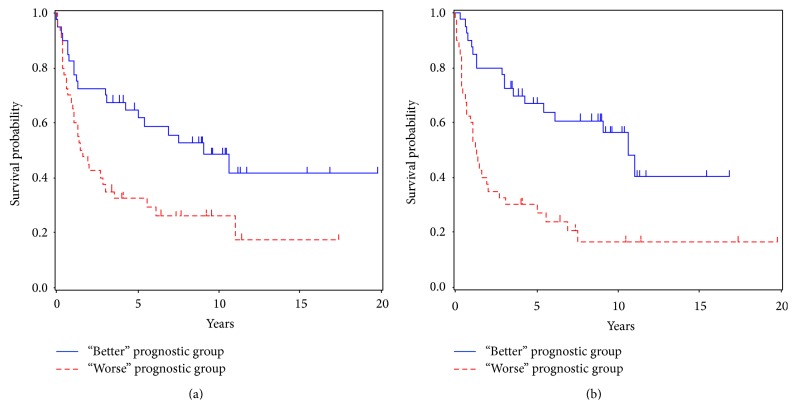
Kaplan-Meier curves of overall survival for “better” and “worse” prognostic groups: (a) the model including 12 genes determined by CV (CV-model) and(b) the model including 42 genes identified by the developed method (42 TP-model).

Table 4 shows the values of the 3 criteria for each model. We found that the values of all 3 criteria for the 42 TP-model were lower than those for the CV-model, suggesting that the model based on the proposed method was more accurate (see Table 4). Additionally, Figure 3 shows that the Kaplan-Meier curves for the 42 TP-model distinguished the “better” and “worse” prognostic groups more definitely than those for the CV-model (42 TP-model, *P* < 0.001; CV-model, *P* = 0.007). Therefore, by using our proposed algorithm, we determined *λ* and were able to select important genes, likely to be correlated with survival, in which the CV was unable to select.

## 4. Discussions

In this study, we proposed an algorithm for estimating the number of TP on the solution path of lasso estimates. Monitoring and determining the number of TP for a series of values *λ* are important because they can increase the probability of uncovering all outcome-predictive genes. The number of TP should be estimated with appropriate accuracy. To confirm the accuracy of our TP, we conducted a simulation study using a typical gene expression dataset. We found that the precision of our algorithm for estimating the number of TP was adequate, although an overestimation occurred with some values of *λ*. However, the overestimation occurred when the true number of TP was saturated, and so it may not cause a problem by passing over genes that truly correlated with survival. In the simulation study where *p*_1_ = 30 and *ρ* = 0.5, the maximum average estimated number of TP was 35.3 at *λ* = 12.4 (see Table 2). Using this *λ* to select TP, an average selection of 29.9 TP within 30 outcome-predictive genes can be made, with the number of TP genes that are passed over being negligible in practice.

The data that have been provided in Table 2 showed that the number of false positives increased, while the number of true positives increased and then plateaued as the tuning parameter decreased. To decrease the number of FP identified while maintaining an adequate number of TP, we should determine the value of *λ* by monitoring both the number of TP and the false positive rate ( = FP/(TP + FP)) in the proposed method.

Additionally, our proposed algorithm was applied to DLBCL data. We determined the value of the tuning parameter based on the maximum number of estimated TP uncovered by the algorithm. We identified 42 TP genes among 96 selected genes based on the ranking of the absolute values of the lasso estimates. We can also identify TP based on model evaluation criteria such as AIC among all possible combinations of 42 genes from 96, that is, _96_C_42_(>10^27^) combinations in total; however, calculation of AIC for all possible gene combinations is a distant approach. To evaluate the efficiency of the approach using the ranking of the lasso estimates, we calculated the AIC for 10,000 randomly chosen models among all the possible models and subsequently compared it with the AIC of our approach. From 10,000 models, the AIC of 425 models (4.25%) was better than that of our approach. This result indicated that our ranking-based approach has a satisfactory performance in practice with respect to the identification of 42 genes. Although investigation of all possible gene combinations is ideal, our approach is a good alternative.

In the application to DLBCL data, in comparison to a CV method by which 12 genes were identified, we identified 42 TP genes with our algorithm, and we improved the prediction accuracy of the model. In practice, some researchers might be satisfied with identifying a few promising genes and would not be unduly worried about passing over others. In such a situation, the CV would be preferable because it developed the model to uncover a few genes with just a small loss of prediction accuracy. However, genes that are selected by the lasso are often investigated with greater scrutiny by genetic researchers, and so passing over outcome-predictive genes by the lasso could represent a major problem. Indeed, if the lasso passes over outcome-predictive genes, some genetic research may not take place. Therefore, when identifying all outcome-predictive genes is a priority, our proposed algorithm will be most useful.

## 5. Conclusions

We developed a method for estimating the number of true positives for a series of values of a tuning parameter in the lasso. We demonstrated the utility of the developed method through a simulation study and an application to a real dataset. Our results indicated that our developed method was useful for determining a value for the tuning parameter in the lasso and reducing the probability of passing over genes that are truly correlated with survival.

## Figures and Tables

**Table 1:** Typical results of gene selection by the lasso.

True condition	The lasso	
	Select	No select
Genes that are not correlated with survival (none-outcome-predictive genes)	False positive (FP)	True negative (TN)

Genes that are truly correlated with survival (outcome-predictive genes)	True positive (TP)	False negative (FN)

**Table 2:** Accuracy of the estimated number of true positives (TP) obtained using the proposed algorithm in the simulation study. Average of a tuning parameter (λ), number of selected genes ($\#\left\{j;{\hat{\beta }}_{j}\left(\lambda \right)\neq 0\right\}$) in the lasso, true number of true positives (True TP), estimated number of TP ($\hat{TP}$), and false positives ($\hat{FP}$) are reported at λ_*k*_ (*k* = 5,10,50,100,150) of the solution path.

*p*_1_	ρ	*k*	λ	$\#\left\{j;{\hat{\beta }}_{j}\left(\lambda \right)\neq 0\right\}$	True TP	$\hat{TP}$	$\hat{FP}$
30	0	5	47.0	5.0	4.4	2.9	2.2
		10	40.8	10.1	8.0	5.8	4.3
		50	22.9	48.6	25.6	28.5	20.1
		100	12.6	86.7	29.9	32.1	54.7
		150	8.6	124.5	30.0	30.7	93.9
	0.5	5	48.6	5.0	4.1	2.8	2.2
		10	42.1	10.0	7.5	5.8	4.2
		50	23.5	48.1	25.2	31.9	16.3
		100	12.4	84.9	29.9	35.3	49.6
		150	8.4	121.2	30.0	31.6	89.6

5	0	5	66.9	5.0	5.0	3.0	2.0
		10	26.3	10.4	5.0	5.2	5.2
		50	17.2	50.1	5.0	5.2	44.9
		100	12.7	93.9	5.0	5.0	88.9
		150	9.8	128.4	5.0	5.0	123.4
	0.5	5	66.8	5.0	5.0	3.0	2.0
		10	26.5	10.3	5.0	5.2	5.1
		50	16.9	49.5	5.0	5.1	44.4
		100	12.4	92.1	5.0	5.0	87.1
		150	9.6	125.2	5.0	5.0	120.2

**Table 3:** GenBank accession numbers and descriptions for 4 genes selected by both CV and the model including the 42 genes identified by the algorithm that we developed.

GenBank accession number	Description
X82240 (AA729003)	T-cell leukemia/lymphoma 1A
AA805575	Thyroxine-binding globulin precursor
LC_29222	—
X59812(H98765)	Cytochrome P450, subfamily XXVIIA polypeptide

**Table 4:** Values of the comparison criteria for the model including 12 genes determined by CV (CV-model) and the model including the 42 genes identified by our developed algorithm (42 TP-model).

Criteria	CV-model	42 TP-model
*P* value of the log-rank test	0.007	<0.001
*P* value for the prognostic index	0.002	<0.001
Deviance	−9.079	−11.297
